# Self‐Adhesive Liquid Metal Channel Patch with Tip‐Guided Conformal Coupling and Leakage Suppression for Skin Bioelectronics

**DOI:** 10.1002/advs.202513259

**Published:** 2025-09-12

**Authors:** Sang‐Woo Lee, Hyeonseok Song, Jinseo Kim, Geonjun Choi, Jaeil Kim, Seongjin Park, Hyejin Jang, Jangho Kim, Hoon Eui Jeong

**Affiliations:** ^1^ Department of Mechanical Engineering Ulsan National Institute of Science and Technology (UNIST) Ulsan 44919 Republic of Korea; ^2^ Department of Convergence Biosystems Engineering Chonnam National University Gwangju 61186 Republic of Korea

**Keywords:** health monitoring, liquid metal electrode, skin electrode, via‐hole interconnects, wearable electronics

## Abstract

Skin‐interfacing electrodes are essential for wearable bioelectronics, yet conventional gel‐ and dry‐type electrodes often suffer from dehydration, poor skin conformity, irritation, and delamination during motion, limiting their long‐term performance. Here, a self‐attachable liquid metal channel (S‐LMC) patch is presented that integrates open‐bottom Galinstan microchannels and micropillar arrays, both featuring re‐entrant geometries for enhances skin adhesion and leakage suppression. A via‐hole interconnect enables direct vertical signal transmission, eliminating the need for bulky wiring and facilitating compact integration. The patch achieves strong, reusable skin adhesion (>60 kPa), low contact impedance (7.35 kΩ·cm^2^ at 10 Hz), and minimal skin irritation. Compared to commercial Ag/AgCl gel electrodes, the S‐LMC patch exhibits >5× lower impedance, >2× higher ECG signal fidelity (20.23 dB vs. 9.03 dB under motion), and >2.4× higher long‐term adhesion after 7 days. Its re‐entrant microarchitecture also improves Galinstan confinement, achieving >2× higher critical pressure for leakage. These features enable motion‐resilient biosignal monitoring and scalable system integration, establishing the S‐LMC patch as a promising platform for next‐generation skin‐conformal bioelectronic interfaces.

## Graphical abstract

A self‐attachable liquid metal channel patch (S‐LMC patch) that integrates tip‐guided micropillars and leakage‐resistant microchannels enables conformal, residue‐free skin interfaces. The patch provides stable and long‐term biosignal acquisition under motion and daily wear, and supports both vertical stacking and lateral expansion, offering a scalable platform for multifunctional, high‐density wearable bioelectronics.

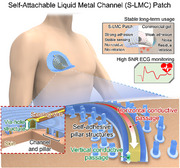

## 1 Introduction

Flexible skin electrodes are widely used in wearable bioelectronic devices for applications such as electrophysiological signal acquisition, disease diagnosis,^[^ ^1^ ^]^ and human–machine interaction.^[^ ^2^ ^]^ High‐fidelity signal acquisition in these systems requires electrodes to maintain intimate contact with the skin, exhibit robust adhesion, ensure long‐term mechanical and electrical stability, and minimize skin irritation and contamination.^[^ ^3^ ^]^ However, simultaneously meeting all of these requirements remains challenging.^[^ ^4^ ^]^ Maintaining reliable contact over extended periods, particularly under dynamic, wet, or dry conditions, is difficult due to the soft, deformable nature of human skin and its complex surface topography.^[^ ^5^ ^]^ Inadequate contact can lead to increased impedance and motion artifacts, degrading signal quality.^[^ ^6^ ^]^ Two primary types of skin‐contact electrodes have been developed to address these issues: wet electrodes and dry electrodes, each offering distinct advantages and limitations.

Wet electrodes, such as Ag/AgCl gel electrodes and conductive hydrogels, use electrolytic gels to improve ionic conductivity and conformal contact, resulting in relatively low skin–electrode impedance.^[^ ^7^ ^]^ However, they suffer from progressive dehydration, leading to increased impedance and reduced signal reliability over time.^[^ ^8^ ^]^ In contrast, dry electrodes employ solid conductive materials (e.g., metal films^[^ ^9^ ^]^ and carbon rubbers^[^ ^10^ ^]^) and offer advantages such as ease of handling, reduced skin irritation, and gel‐free operation. However, traditional dry electrodes based on rigid structures often suffer from mechanical mismatch with soft skin tissues, which limits conformability and increases motion‐induced artifacts and interfacial impedance due to lower conductivity and adaptability.^[^ ^11^ ^]^ To address these limitations, recent developments have introduced soft and nanomaterial‐based dry electrodes (e.g., graphene,^[^ ^12^ ^]^ conductive polymers,^[^ ^13^ ^]^ or silver nanowires^[^ ^14^ ^]^) with enhanced mechanical compliance and improved skin conformity. These advances have contributed to more stable signal acquisition during movement, making them promising alternatives to conventional designs.^[^ ^15^ ^]^ Nonetheless, even these advanced systems typically exhibit lower conductivity than liquid metal or hydrogel‐based wet electrodes.^[^ ^16^ ^]^ In addition, their relatively weak and non‐reusable adhesion often requires external tapes or fixation strategies, reducing integration simplicity and limiting long‐term usability in motion‐rich environments.^[^ ^17^ ^]^

Gallium‐based liquid metals, such as eutectic gallium–indium (EGaIn) and Galinstan, have emerged as promising alternatives due to their high conductivity,^[^ ^18^ ^]^ low vapor pressure,^[^ ^19^ ^]^ and deformability,^[^ ^20^ ^]^ and biocompatibility.^[^ ^21^ ^]^ These properties allow for intimate, long‐lasting contact with rough skin surfaces, making them attractive for wearable sensing. However, their high fluidity presents challenges: the liquid metal (LM) may escape confinement under pressure, contaminate the skin, and cause electrical shorting.^[^ ^22^ ^]^ To mitigate this, liquid metals are often embedded in composites, but this reduces conductivity and pattern resolution. They also lack intrinsic adhesion.^[^ ^23^ ^]^ To address these limitations, bioinspired microstructured adhesives have been developed, mimicking the reversible attachment mechanisms of biological systems.^[^ ^24^ ^]^ These dry adhesives, typically composed of micropillars, achieve conformal and reversible contact through van der Waals interactions.^[^ ^25^ ^]^ They provide skin‐friendly, repeatable adhesion without chemical glues.^[^ ^26^ ^]^ However, their non‐conductive nature and lack of integrated signal pathways prevent their direct use as skin electrodes, necessitating hybrid designs that combine adhesion and electrical functionality.

Herein, we present a S‐LMC patch that combines open‐bottom microfluidic channels filled with Galinstan and a micropillar‐based adhesive interface, both featuring re‐entrant geometries to enable robust, residue‐free skin contact and effective liquid metal confinement. The re‐entrant tips of the micropillars and channel walls suppress leakage under pressure while enhancing skin adhesion through conformal mechanical interlocking. A centrally aligned via‐hole enables direct vertical signal transmission, eliminating bulky wiring and supporting compact circuit integration. This integrated architecture yields a conformal, low‐impedance, and motion‐resilient electrical interface with strong and reusable adhesion (>60 kPa), low contact impedance (7.35 kΩ·cm^2^ at 10 Hz), and high ECG signal fidelity (SNR of 26.8 dB). Compared to commercial Ag/AgCl gel electrodes, the S‐LMC patch demonstrates >5× lower impedance, and >2.4× higher long‐term adhesion after 7 days. These combined features enable long‐term, high‐fidelity biosignal monitoring even under mechanical deformation.

## 2 Results and Discussion

### 2.1 Design of the S‐LMC Patch

**Figure 1:**
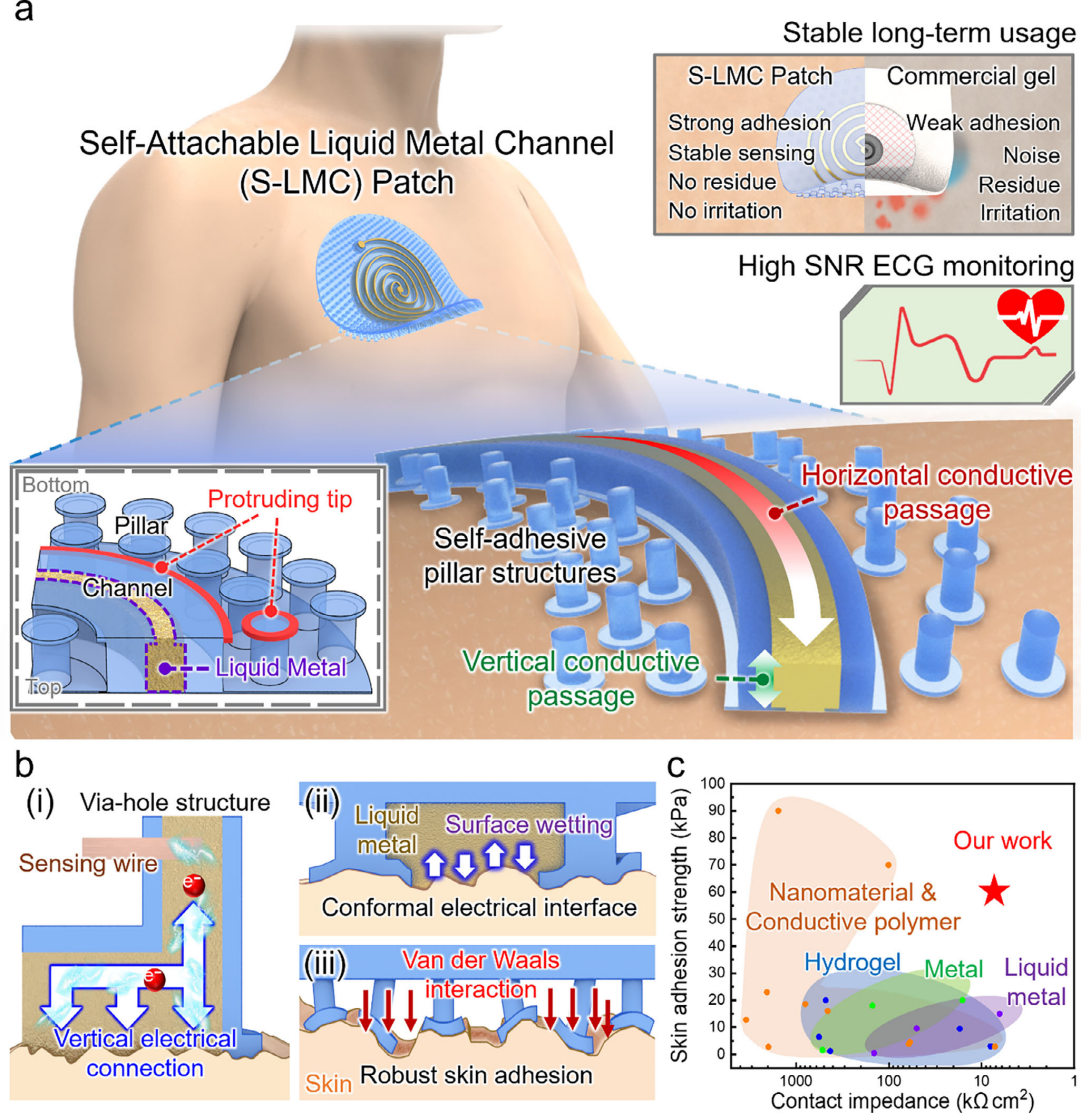
Design and key advantages of the S‐LMC patch. a) Schematic of the S‐LMC patch architecture, integrating an open‐bottom microfluidic channel and micropillar‐based adhesive interface, with a performance comparison to a commercial gel electrode. b) Functional features of the S‐LMC patch: (i) vertical signal transmission via centrally aligned via‐hole; (ii) conformal contact and low‐impedance coupling enabled by liquid metal wetting; (iii) robust, reversible adhesion via van der Waals interactions at micropillar tips. c) Benchmark comparison of skin adhesion strength and electrical impedance versus conventional dry, hydrogel, and liquid metal electrodes.

The S‐LMC patch is rationally engineered to deliver a low‐impedance, leakage‐resistant, residue‐free, and skin‐conformal electrical interface with robust yet reversible adhesion—a combination of features not achievable with commercial gel electrodes (**Figure** **1**a). The patch integrates an open‐bottom microfluidic channel filled with Galinstan, a highly conductive liquid metal, and a surrounding array of micropillars. These micropillars feature bioinspired protruding edge tips—outward‐extending structures along the pillar head perimeter that resemble mushroom cap brims—to enhance skin conformability and adhesion. This architecture enables both stable signal transduction and clean, irritation‐free skin contact during prolonged use.

The microchannel, embedded within the patch, guides Galinstan laterally along the skin to form a continuous conductive path. Capillary forces confine the liquid metal within the channel, while the protruding tips partially overhang it to mechanically anchor the Galinstan and suppress leakage under pressure or deformation (Figure 1a, inset). At the center of the patch, a via‐hole structure is directly aligned beneath the channel, forming a vertical electrical pathway from the skin interface through the liquid metal to external sensing components (Figure 1b‐i). This dual‐mode signal routing—lateral conduction along the channel and vertical transmission through the centrally positioned via‐hole—ensures compact, low‐resistance, and uninterrupted signal delivery.

To ensure efficient charge transfer, the patch employs a tip‐guided interface that promotes conformal wetting of Galinstan against the skin microtexture (Figure 1b‐ii). This minimizes interfacial resistance and enhances electrical coupling across the entire contact area. In parallel, the surrounding micropillars enhance mechanical adhesion (Figure 1b‐iii). The protruding tips amplify local van der Waals interactions and increase effective contact area, leading to strong yet peelable adhesion. The discrete geometry of the micropillars uniformly distributes interfacial stress, preserving attachment under motion and deformation.^[^ ^24a^ ^]^

This structurally integrated approach supports both mechanical adaptability and high‐fidelity electrical functionality, even under dynamic conditions such as bending, twisting, or repeated application cycles. Benchmark comparisons in Figure 1c and Table S1 (Supporting Information) confirm the advantages of the S‐LMC patch, which simultaneously achieves significantly higher adhesion strength (>60 kPa) and markedly lower contact impedance (≈7.35 kΩ·cm^2^ at 10 Hz) compared to conventional dry, hydrogel, or liquid metal electrodes.

### 2.2 Fabrication and Structure of the S‐LMC Patch

**Figure 2:**
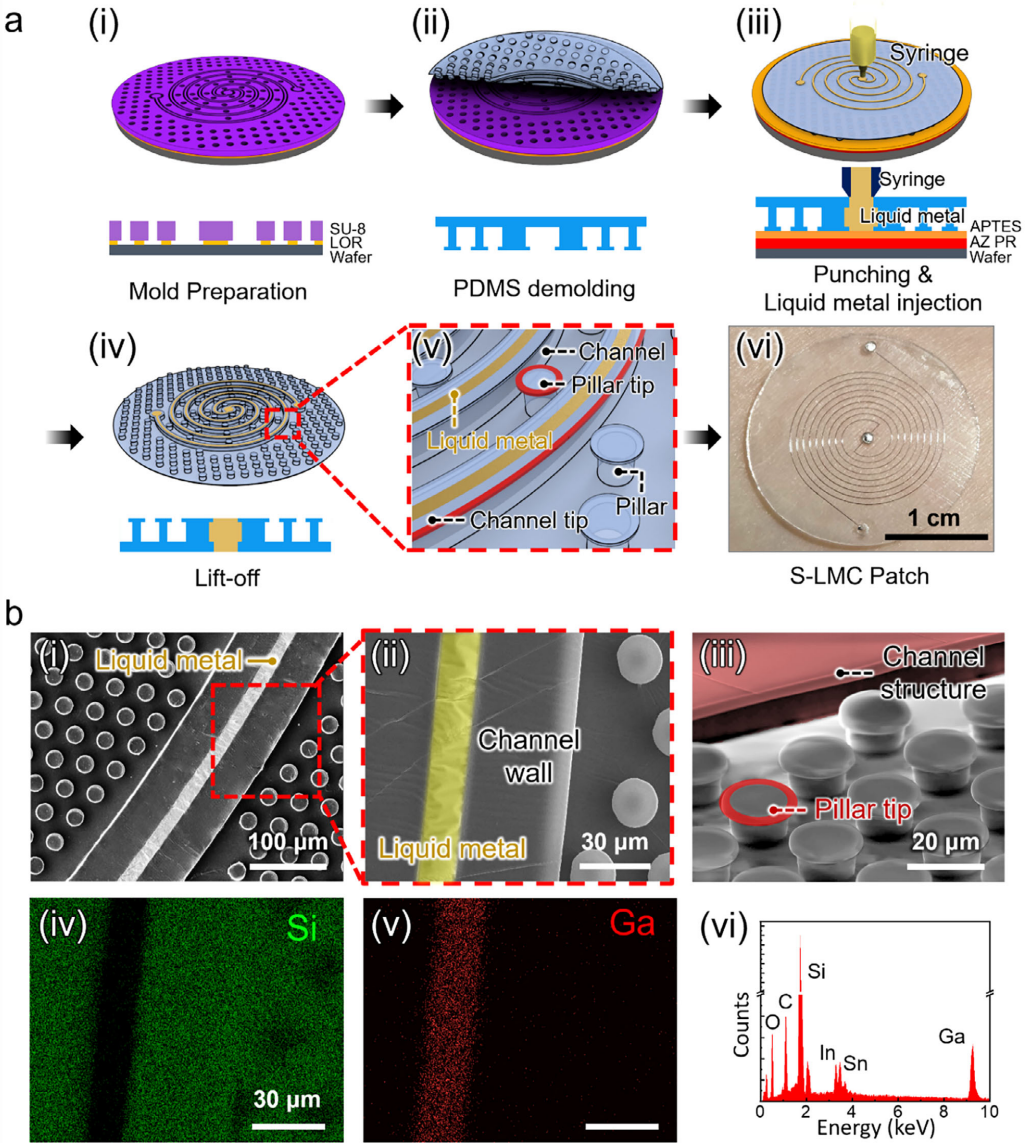
Fabrication and structural characterization of the S‐LMC patch. a) (i‐v) Schematic of the fabrication process and (vi) photograph of the fabricated S‐LMC patch. b) (i) SEM image of the microchannel and surrounding micropillar array; (ii) magnified view showing liquid metal confinement within the channel; (iii) SEM of microtip geometry at the pillar top; (iv, v) Elemental maps of Si and Ga, confirming selective localization of Galinstan within the channel; (vi) EDS spectrum indicating the presence of Ga, In, and Sn from the Galinstan filler.

**Figure** **2**a presents the fabrication process of the S‐LMC patch via replica molding using polydimethylsiloxane (PDMS) and a photolithographically patterned silicon master mold. The mold was designed with negative patterns of the microchannel and micropillar array using a bilayer photoresist system (see Experimental Section for details). After PDMS casting and curing, the molded patch was demolded to yield a flexible structure with open microchannels and self‐adhesive micropillars. Three access holes were subsequently perforated to serve as inlet, outlet, and via‐hole interconnects. Galinstan, a eutectic gallium–indium–tin alloy, was then injected into the microchannels through the inlet using a syringe. Protruding tip structures were concurrently formed on both the micropillars and the channel walls during the molding process. These features along the inner and outer channel surfaces enhance wetting while anchoring the Galinstan to prevent leakage. The resulting S‐LMC patch demonstrates successful and selective confinement of Galinstan exclusively within the microchannel regions (Figure 2a–vi).

Figure 2b shows structural and compositional characterization of the S‐LMC patch. The SEM image in Figure 2b‐i confirms the successful formation of spiral microchannels (20 µm inner width, 40 µm wall thickness, 20 µm height) surrounded by a dense array of micropillars (28 µm outer diameter, 20 µm stem diameter, 4 µm tip length, 3 µm tip thickness, 20 µm height, and 30 µm pitch). The protruding tips are clearly visible along both the channel sidewalls and micropillars, as highlighted in the magnified view (Figure 2b‐ii,iii). These microscale geometries are engineered to optimize both adhesive contact and Galinstan retention. The bright contrast along the channel center in SEM indicates the presence of Galinstan, which functions as a highly conductive, skin‐conformal interconnect. To verify its selective localization, EDS analysis was conducted on the channel region. Elemental mapping images (Figure 2b‐iv,v) further confirm the spatial separation of Ga and silicon (Si), with Ga confined within the channel and Si distributed across the PDMS matrix. The spectrum (Figure 2b‐vi) reveals prominent peaks for gallium (Ga), indium (In), and tin (Sn), consistent with Galinstan composition. This compositional distinction confirms that the liquid metal is precisely localized within the intended conductive region without infiltrating the surrounding adhesive structures. Combined with the micropillar‐based adhesive architecture, this enables clean, conformal, and mechanically stable skin contact, ensuring both electrical performance and durable skin adhesion under prolonged and dynamic use.

### 2.3 Skin Adhesion Performance of the S‐LMC Patch

**Figure 3:**
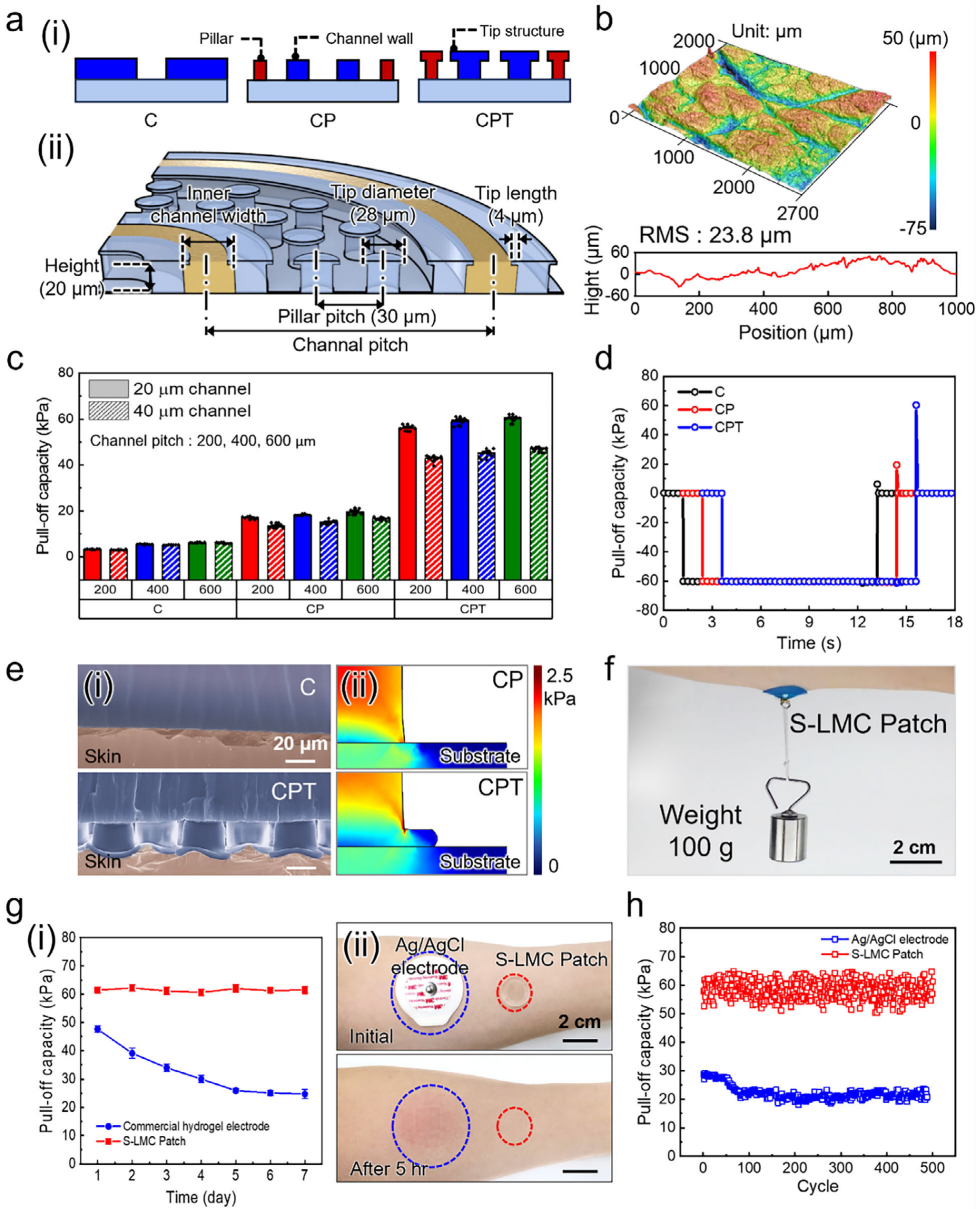
Adhesion performance and skin compatibility of the S‐LMC Patch. a) (i) Schematic comparison of C, CP, and CPT structures; (ii) Schematic illustration of the CPT architecture, highlighting tip dimensions and channel layout. b) 3D surface profile and RMS roughness of porcine skin. c) Pull‐off strength of S‐LMC patches with varying geometries (channel width and pitch): n = 7 per group. d) Pull–off strength curves comparing detachment resistance of C, CP, and CPT patches. e) (i) Cross‐sectional SEM of skin contact; (ii) FEA of stress distribution beneath CP and CPT. f) Self‐adhesion demonstration using a 100 g suspended load. g) (i) Pull‐off strength over 7 days, n = 7 independent samples; (ii) Skin irritation after 5 h attachment. h) Adhesion repeatability over 500 attachment–detachment cycles.

The skin adhesion performance of the S‐LMC patch was evaluated using porcine skin as a substrate. To elucidate the role of structural design, three distinct configurations were fabricated: channel only (C), channel with pillars (CP), and channel with pillars incorporating tip structures (CPT), with defined geometric parameters including channel width and channel pitch (**Figure** **3**a). The micropillars had an outer diameter of 28 µm, a height of 20 µm, and were arranged with a 30 µm pitch, except within the channel regions. To evaluate the skin surface morphology, 3D laser confocal microscopy was performed, revealing a root‐mean‐square (RMS) roughness of approximately 23.8 µm and pronounced topographical irregularities (Figure 3b).

Under a preload of 60 kPa and a retraction rate of 1 mm s^−1^, the CPT sample demonstrated a maximum pull‐off strength of 60.3 kPa—approximately 10× higher than C (6.2 kPa) and over 3× higher than CP (19.4 kPa) (Figure 3c). To evaluate rate‐dependence, additional tests at slower retraction speeds (0.1 and 0.5 mm s^−1^) were conducted, confirming consistent adhesion performance regardless of detachment rate (Figure S1, Supporting Information). Among identical sample types, configurations with narrower channel widths (20 µm) and wider pitches (600 µm) exhibited higher adhesion than those with wider channels (40 µm) or denser spacing (200–400 µm), attributed to the increased number of discrete micropillars. Contact area analysis confirmed that adhesion strength correlated more strongly with the pillar contact area rather than the total contact area (Figure S2, Supporting Information), consistent with prior findings that discrete micropillar structures provide superior adhesion compared to continuous or grid‐like patterns.^[^ ^27^ ^]^ Based on these findings, a channel width of 20 µm and a pitch of 600 µm were selected as the optimized S‐LMC patch configuration.

Pull‐off strength measurements (Figure 3d) confirmed the superior detachment resistance of the CPT structure. Cross‐sectional SEM analysis (Figure 3e) revealed that CPT structures formed intimate, void‐free contact with the rough skin surface, attributed to the bioinspired pillar design that enabled conformal mechanical engagement with surface asperities. Finite element analysis further indicated that CPT structures provided a more uniform stress distribution across the contact interface than CP designs. The combined effects of enhanced conformity, tip‐induced stress homogenization, and increased contact area explain the markedly improved adhesion of the CPT architecture. To investigate the influence of liquid metal, CPT patches were tested with and without Galinstan injection. The adhesion strength remained nearly unchanged (60.7 kPa without Galinstan and 60.3 kPa with Galinstan; Figure S3, Supporting Information), indicating that adhesion is predominantly governed by the surface microarchitecture rather than the liquid metal itself.

The practical self‐adhesion of the S‐LMC patch was demonstrated by suspending a 100 g weight using a 2 cm‐diameter patch adhered to the forearm (Figure 3f). The strong self‐adhesion of the S‐LMC patch was demonstrated by suspending a 100 g weight using a 2 cm‐diameter patch adhered to the skin (Figure 3f). The patch detached after 30 minutes under vertical loading but remained securely attached under lateral shear for over 10 hours, highlighting its robust load‐bearing capacity. Long‐term stability was evaluated over 1–7 days and benchmarked against a commercial hydrogel electrode (Figure 3g‐i). Notably, after extended skin attachment, the Ag/AgCl electrode induced visible irritation, whereas the S‐LMC patch did not (Figure 3g‐ii). This contrast arises from differences in adhesion and interface design: the commercial electrode employs acrylate adhesives and an occlusive backing that disrupts the stratum corneum and trap moisture, causing irritation. In contrast, the S‐LMC patch uses an adhesive‐free, breathable micropillar structure that conforms to the skin via van der Waals interactions, thereby minimizing skin irritation.^[^ ^28^ ^]^ In vitro cytotoxicity testing further confirmed the patch's biocompatibility, showing >95% cell viability (Figure S4, Supporting Information). These findings align well with previous reports.^[^ ^[Link]^, ^[Link]^ ^]^ The hydrogel's adhesion deteriorated from 47.7 kPa to 24.7 kPa due to dehydration, while the S‐LMC patch consistently maintained adhesion above 60 kPa, benefiting from its mechanically robust microarchitecture and stable Galinstan confinement. Moreover, extended wear tests showed no skin irritation with the S‐LMC patch, unlike the Ag/AgCl electrode, which induced visible marks and redness after 5 hours, highlighting its superior skin compatibility. Reusability was assessed over 500 attachment–detachment cycles. The S‐LMC patch retained consistent adhesion, whereas the commercial electrode exhibited a marked decline (Figure 3h). These results establish the S‐LMC patch as a durable, skin‐friendly, and reusable adhesive platform well‐suited for long‐term epidermal electronics.

### 2.4 Leakage Suppression Enabled by Re‐Entrant Tip Geometry

**Figure 4:**
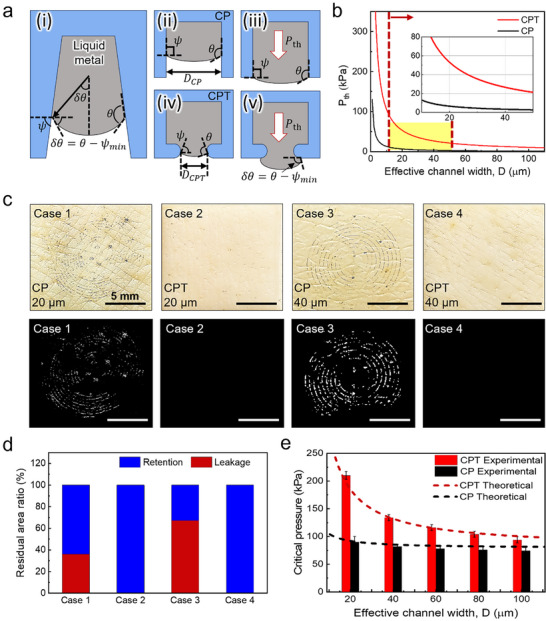
Leakage Suppression performance of the S‐LMC Patch. a) Conceptual schematics defining: (i) key geometric parameters of microchannels: (ii, iii) CP structure; (iv, v) CPT structure. b) Comparison of *P_th_* values between CP and CPT structures as a function of effective channel width. c) Surface residue observed on porcine skins after external pressure application and detachment. d) Quantitative analysis of leaked area normalized to channel size for each configuration. e) Comparison of experimentally measured and theoretically predicted *P_critical_* for CP and CPT structures as a function of effective channel width, n = 7 independent samples.

The re‐entrant tip geometry of the S‐LMC patch plays a pivotal role in stabilizing the confined LM interface and suppressing leakage under mechanical pressure.^[^ ^29^ ^]^ To understand this behavior, we adopted an interfacial stability model that describes the pressure‐induced distortion of the LM–air interface within microchannels (**Figure** **4**a). A key parameter governing this behavior is the local wall angle, *ψ*, defined as the angle between the channel wall and the horizontal plane. Geometrically, *ψ* describes the tapering of the wall near the channel tip: *ψ* ≈ 90° for vertical walls (CP design), and *ψ* → 0° for re‐entrant profiles (CPT design).^[^ ^30^ ^]^

Under increasing internal pressure, the LM interface bulges outward with curvature *δθ = θ – ψ*, where *θ* is the intrinsic contact angle of the LM. When *ψ* reaches a minimum critical value (*ψ_min_*), the interface becomes unstable and leakage occurs (Figure 4a‐ii–v). The theoretical interfacial pressure required to trigger this instability (*P_th_*) is expressed as (see Note S1, Supporting Information for more details):^[^ ^29^ ^]^

(1) $$P_{th}=\frac{2\gamma_{lv}\sin\left(\theta -\psi_{min}\right)}{D}$$

where *γ_lv_* represents the liquid‐vapor surface tension and *D* is the effective channel width. This equation indicates that reducing *ψ_min_* or *D* increases the resistance to leakage. In CPT geometry, *ψ_min_* approaches zero, resulting in substantially higher *P_th_* and improved leakage suppression, as supported by predictions in Figure 4b.

To experimentally validate this theory, we performed leakage tests using porcine skin‐mounted S‐LMC patches under a uniform external pressure of 120 kPa, across four configurations: CP or CPT channels at widths of 20 µm or 40 µm (Cases 1–4, Figure 4c). LM leakage occurred in both CP cases, especially Case 3 (40 µm), while CPT designs (Cases 2 and 4) showed no leakage. Quantification of residual LM area (Figure 4d) further confirmed the superior stability of the CPT design.

To assess geometric scalability, the critical pressure (*P_critical_*) —defined as the externally applied pressure at the patch backside at which LM leakage is first observed—was measured for channel widths ranging from 20 to 100 µm (Figure 4e). This value differs from the theoretical threshold pressure (*P_th_*), which describes interfacial instability within an idealized, non‐deformable channel. ​The CPT design consistently exhibited higher *P_critical_* than CP across all channel sizes, particularly at narrower widths where geometric confinement dominates.

To approximate *P_critical_* from the model, we introduced an empirical correction incorporating the skin's reaction pressure (*P_r_*), estimated from Hertzian contact approximation (see Note S2, Supporting Information), along with a scaling factor *α*:^[^ ^31^ ^]^

(2) $$P_{critical}=\alpha \left(P_{th}+P_{r}\right)$$

Here, *α* = 2 is a fitted parameter that aligns the model with observed leakage thresholds. This correction accounts for unmodeled factors such as nonlinear deformation, substrate compliance, and microscale structural effects. The modified equation showed good agreement with the experimental results (Figure 4e), demonstrating that the re‐entrant profiles (CPT design) significantly enhance the mechanical stability of LM‐filled microchannels.

### 2.5 Electrical Performance of the S‐LMC Patch

**Figure 5:**
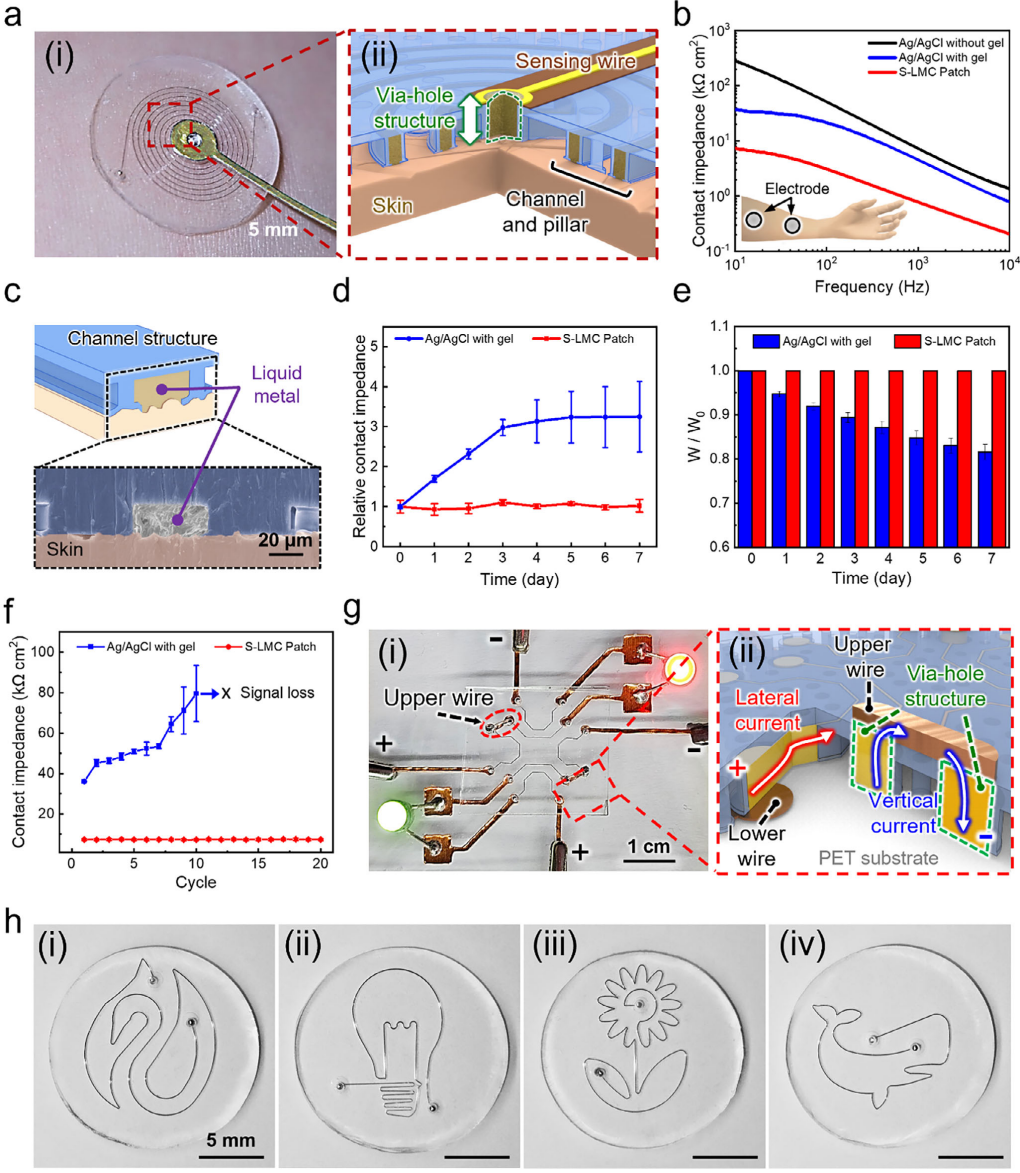
Electrical performance and system integration of the S‐LMC patch. a) Optical image of the patch attached to the forearm with via‐hole–connected sensing wire; schematic showing horizontal microchannels and vertical via‐hole for bidirectional signal routing. b) Contact impedance comparison between S‐LMC and commercial Ag/AgCl electrodes over 10^1^–10⁴ Hz. c) Schematic and SEM image showing conformal contact between Galinstan and skin. d) Relative contact impedance over 7 days under ambient conditions, n = 7 independent samples. e) Weight retention of each patch, indicating dehydration resistance, n = 3 independent samples. f) Impedance stability over 20 attachment–detachment cycles, n = 7 independent samples. g) Photograph of LED circuit driven by the S‐LMC patch (left); schematic of lateral and vertical conduction pathways (right); h) Spatially controlled LM microelectrode configurations enabled by structural confinement within microchannels.

Reliable electrical performance in skin‐interfaced electrodes demands strong adhesion and conformal contact to minimize interfacial impedance and ensure signal fidelity. Unlike conventional dry electrodes, which often separate adhesive and conductive regions, the S‐LMC patch integrates microtip‐structured adhesive pillars with embedded liquid metal channels, enabling both stable mechanical attachment and efficient electrical coupling (**Figure** **5**a‐i). A schematic of the device architecture (Figure 5a‐ii) illustrates how the liquid metal interfaces with the skin while a via‐hole interconnect routes signals vertically to external electronics, allowing for bidirectional (in‐plane and out‐of‐plane) signal transmission without compromising skin conformity. This configuration also facilitates straightforward electrical integration with external devices, eliminating the need for bulky wiring or complex interface circuits.

The S‐LMC patch exhibited consistently lower skin–electrode impedance across the entire frequency spectrum (10^1^–10⁴ Hz) compared to commercial Ag/AgCl gel electrodes (Figure 5b). At 10 Hz, the measured impedance was 7.35 kΩ·cm^2^—over five times lower than the gel electrode—underscoring the superior electrical interface enabled by the conformal Galinstan–skin contact and the intimate coupling promoted by tip‐guided wetting (Figure 5c). Here, the microtip structures serve dual roles: they suppress LM dewetting by confining the Galinstan along skin micro‐contours, and they redistribute interfacial stress to mitigate delamination. These combined effects allow the high‐surface‐tension LM to achieve conformal, stable contact even on topographically irregular surfaces, thereby sustaining low impedance (Figure S5, Supporting Information).

Long‐term stability under ambient conditions further confirmed the robustness of the electrical interface. Over a 7‐day period, the S‐LMC patch preserved its initial impedance with negligible drift, whereas the commercial hydrogel electrode exhibited progressive impedance degradation due to dehydration (Figure 5d). Corroborating this, weight loss measurements revealed that the Ag/AgCl electrode—applied with 100 mg of Signa Gel—lost more than 18% of its mass, in contrast to the negligible mass change observed for the Galinstan‐filled S‐LMC patch, which benefits from the liquid metal's low vapor pressure (Figure 5e). Mechanical reliability was evaluated through repeated attachment‐detachment cycling. After 20 cycles, the S‐LMC patch maintained stable impedance with no measurable degradation, while the commercial electrode experienced a sharp rise in impedance after just 10 cycles due to adhesive delamination and drying (Figure 5f). These findings confirm the patch's suitability for long‐term and reusable skin applications.

To validate functional integration into real circuits, we fabricated electrode arrays with via‐hole–connected external wires. As shown in Figure 5g, the S‐LMC patch successfully powered multiple LEDs via both lateral and vertical conduction pathways, demonstrating uninterrupted current delivery through a single‐layered platform. To validate functional integration into real circuits, we fabricated electrode arrays with via‐hole–connected external wires. As shown in Figure 5g, the S‐LMC patch successfully powered multiple LEDs via both lateral and vertical conduction pathways, demonstrating uninterrupted current delivery through a single‐layered platform. Unlike conventional systems that require stacked layers or rigid interconnects,^[^ ^32^ ^]^ the LM‐filled via‐holes form conformal and continuous vertical pathways within a soft monolithic structure, eliminating the need for external interconnect wires and preserving mechanical flexibility.^[^ ^33^ ^]^

In parallel, embedded LM microchannels ensure reliable lateral conduction with high mechanical compliance and deformability, unlike composite conductors or serpentine‐patterned metal films which are susceptible to fatigue and resistance drift under repeated deformation.^[^ ^34^ ^]^ This dual‐pathway architecture simplifies circuit layout and enables the formation of multiple, spatially resolved, and independently addressable electrodes within a compact and deformable platform. Additionally, photolithography‐based patterning allows precise control over microchannel geometries (Figure 5h), structurally guiding the liquid metal to maintain well‐defined electrode boundaries and suppress lateral spreading, further supporting advanced bioelectronic integration.

### 2.6 ECG Monitoring Application of the S‐LMC Patch

**Figure 6:**
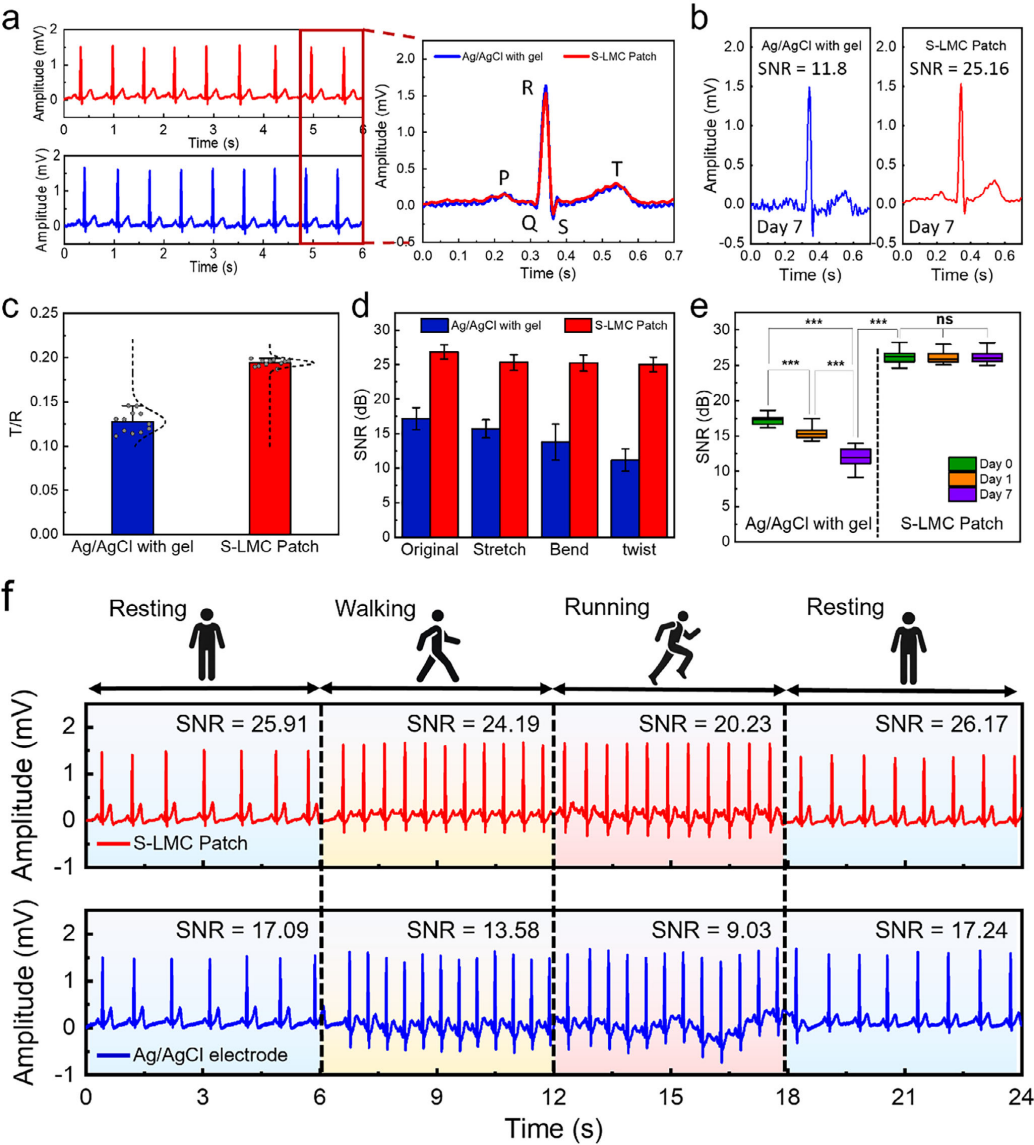
ECG monitoring performance of the S‐LMC Patch. a) Representative ECG signals recorded using the S‐LMC patch (red) and commercial Ag/AgCl electrode (blue) under static conditions. b) Comparison of waveform quality after 7‐day continuous attachment. c) T/R peak amplitude ratios under static conditions. d) Signal‐to‐noise ratio (SNR) under stretching, bending, and twisting. e) SNR variation over 7 days of ambient exposure. f) ECG signals and corresponding SNRs recorded during resting, walking, and running. Each graph represents n = 12 independent samples.

The S‐LMC patch combines strong skin adhesion, low interfacial impedance, and long‐term mechanical stability to enable reliable, high‐fidelity biosignal monitoring. To assess its practical applicability, we performed electrocardiogram (ECG) measurements using a standard limb‐lead configuration (electrodes on both wrists and a reference on the right leg) under both static and dynamic conditions. **Figure** **6**a shows representative ECG waveforms obtained using the S‐LMC patch and a commercial Ag/AgCl electrode under static conditions. After 7 days of continuous skin attachment (Figure 6b), the commercial electrode exhibited signal degradation, characterized by increased background noise and reduced waveform clarity. This degradation is quantitatively supported by a decline in its SNR from 20.3 dB (Day 0) to 11.8 dB (Day 7). In contrast, the S‐LMC patch retained well‐defined waveforms with high SNR (26.8 to 25.16 dB), indicating superior long‐term recording stability. To further assess signal quality, we evaluated the T/R amplitude ratio, a clinically relevant index of repolarization and arrhythmia detection. As shown in Figure 6c, the S‐LMC patch achieved a T/R ratio of 0.20, substantially higher than the 0.13 recorded by the commercial electrode, validating enhanced repolarization peak resolution.

The robustness of the S‐LMC patch under mechanical deformation was assessed by measuring signal‐to‐noise ratio (SNR) during skin deformation modes including stretching, bending, and twisting (Figure 6d). The patch maintained high and stable SNRs (>25 dB) across all deformation modes, whereas the commercial electrode exhibited substantial noise and variability. This performance is attributed to the high electrical conductivity of the LM and the conformal skin contact achieved through tip‐guided mechanical confinement, which together stabilize the electrode–skin interface and ensure uniform wetting of microtextured skin—thereby reducing contact noise and enhancing SNR. Long‐term electrical performance was further examined over 7 days of ambient wear (Figure 6e). Although mild reduction in adhesion was observed under moist conditions (Figure S6, Supporting Information), the micropillar‐based structure of the S‐LMC patch maintained effective skin contact throughout prolonged use. As a result, the patch preserved stable SNRs over the 7‐day period, while the Ag/AgCl electrode showed gradual degradation, which highlights the S‐LMC patch's superior environmental and mechanical resilience. Ultimately, the patch retained sufficient contact stability to enable high‐fidelity signal acquisition during prolonged wear.

Finally, real‐time ECG signals were recorded during physical activities including resting, walking, and running (Figure 6f). The S‐LMC patch reliably tracked heart activity across all states, maintaining waveform fidelity and high SNR, whereas the commercial electrode suffered from motion‐induced artifacts and diminished SNR, especially under dynamic conditions. These results confirm the S‐LMC patch's suitability for continuous, motion‐resilient physiological monitoring in wearable healthcare applications.

## 3 Conclusion

In summary, we have developed a structurally integrated S‐LMC patch that combines tip‐guided adhesive microstructures with leakage‐resistant liquid metal microchannels, enabling high‐performance skin‐conformal electronics. Through its hybrid design, the patch achieves strong and durable skin adhesion (pull‐off strength over 60 kPa, >500 reuse cycles), along with consistently low interfacial impedance (7.35 kΩ·cm^2^ at 10 Hz) and high signal fidelity (SNR > 25 dB) over 7 days of use. These properties are sustained under mechanical deformation and long‐term ambient exposure, highlighting its robustness for practical applications.

Key to this performance is the synergistic combination of microtip‐structured adhesion and the conformal wetting behavior of the liquid metal interface, which together minimize impedance fluctuation and maintain stable contact under motion or prolonged wear. The microfabrication approach further supports scalable customization of high‐density, multichannel electrode architectures, while via‐hole electrical routing facilitates integration with external circuits and wearable devices. Functional demonstrations, including multi‐LED activation and continuous ECG monitoring during rest and motion, validate the platform's capacity for reliable signal acquisition across diverse physiological conditions. Collectively, these results position the S‐LMC patch as a promising foundation for next‐generation skin‐interfaced electronics, offering a rare combination of mechanical durability, electrical stability, and interface adaptability required for long‐term biosignal monitoring and multimodal wearable healthcare applications.

## 4 Experimental Section

### Fabrication of the S‐LMC Patch

The S‐LMC patch was fabricated using a multi‐step soft lithography process. A bilayer master mold with microchannels and tip‐terminated micropillars was prepared on a Si wafer using sequential spin‐coating of lift‐off resist (LOR 30 B, Microchem, USA) and photoresist (SU‐8 3010, Microchem, USA). The LOR and SU‐8 layers were soft‐baked at 200 °C for 30 min and 95 °C for 10 min, respectively. UV exposure (365 nm, 150 mJ cm^−2^) through a photomask defined the SU‐8 microstructures, followed by development in SU‐8 developer for 5 min. A 4‐µm undercut was created in the LOR layer using AZ 400 K (AZ Electronic Materials, USA). PDMS prepolymer (Sylgard 184, Dow, USA; 5 wt% curing agent) was poured onto the master mold and cured at 70 °C for 1.5 h. This ratio was chosen to enhance skin adhesion while ensuring reliable microstructure fabrication. The cured PDMS was plasma‐treated (100 W, 3 min; CUTE, Femto Science, Korea), then coated with a 10 wt% dextran solution (Sigma‐Aldrich, USA) and baked at 120 °C for 1 min to form a sacrificial layer. An additional PDMS layer was cast and cured on top.

Holes were perforated at both ends of the channel for Galinstan injection. To form an open‐bottom channel structure, positive photoresist (AZ4330, Microchem, USA) was spin‐coated onto a Si wafer and soft‐baked for 1 min, followed by plasma activation (100 W, 3 min). APTES (Sigma‐Aldrich, USA) was deposited via vapor‐phase silanization at 90 °C for 2 h. The PDMS structure was bonded via plasma‐assisted bonding (100 W, 1.5 min), and Galinstan (Thermo Fisher Scientific, USA) was injected using a syringe. Excess liquid metal was removed with 1 M HCl. The dextran sacrificial layer was dissolved with D.I. water, and the final release was achieved by dissolving the photoresist in ethanol over 24 h.

### Pull‐Off Adhesion and Residue Characterization

Pull‐off adhesion tests were performed using a custom‐built tester comprising a substrate holder and a vertically actuated jig (Figure S7, Supporting Information). Target substrates were affixed to the holder using double‐sided tape (3M, USA), while adhesive samples—either a commercial gel (1.5 cm diameter, 3M, USA) or the S‐LMC patch (2 cm diameter)—were mounted on the downward‐facing surface of the jig, also secured with double‐sided tape. Each sample was brought into contact with porcine skin substrates under a controlled preload of 60 kPa. The jig was then retracted vertically at a constant rate of 1 mm s^−1^ until detachment occurred. Adhesive forces during this process were recorded using a load cell (Ktoyo, Republic of Korea) connected to the jig. Post‐detachment, the porcine skin was visually inspected to assess residual liquid metal transfer from the S‐LMC patches. Residue areas were quantified using ImageJ software (NIW, USA), providing a comparative measure of interfacial integrity and leakage potential upon removal.

### Surface Analysis

Scanning electron microscopy (SEM) and energy‐dispersive X‐ray spectroscopy (EDS) were conducted using an S‐4800 microscope (Hitachi, Japan) to characterize the microchannels, micropillars, and cross‐sections of the microchannel structures. Prior to imaging, samples were coated with a 5 nm‐thick platinum (Pt) layer using a K575X sputter coater (Quorum Emitech, UK) to enhance conductivity. The surface topography of porcine skin was examined using a 3D laser confocal microscope (VK‐X3050, Keyence, Japan), and surface roughness parameters were extracted from the scanned images using VK‐A3E image analysis software.

### Electrical Characterizations

Skin contact impedance of the S‐LMC patch was measured using an LCR meter (E4980A, Keysight, USA) with two electrodes placed 5 cm apart on the forearm. Test electrodes included Ag/AgCl electrodes (2223H, 3M, USA) with and without conductive gel (Signa Gel, Parker Labs, USA), as well as S‐LMC patches. Impedance was recorded across a frequency range of 10^1^ to 10⁴ Hz. To assess long‐term stability, relative contact impedance and weight loss of Ag/AgCl electrodes (with gel) and S‐LMC patches were evaluated under ambient conditions (40% RH, room temperature) over 7 days. Contact impedance was measured using the same setup, and impedance changes were tracked over time. Weight loss was monitored by measuring sample mass at predetermined intervals with an analytical balance, and relative weight change was calculated as a percentage of the initial weight.

### ECG Signal Measurement

ECG signals were acquired using a commercial ECG module (AD8232, Analog Devices Inc., USA) interfaced with an Arduino Uno development board. Three electrodes were used for signal acquisition: two placed on the wrists and a ground electrode on the right ankle. Both Ag/AgCl electrodes and S‐LMC patches were tested under identical conditions. The digitized ECG signals were transmitted to a laptop and processed using MATLAB. SNR was calculated to compare the signal quality of each electrode type using the equation:

(3) $$SNR\left(dB\right)=20\log_{10}\times \frac{A_{signal}}{A_{noise}}$$

where *A_signal_* represents the root‐mean‐square (RMS) amplitude of all R‐peaks in the notch‐filtered ECG signal, and *A_noise_* corresponds to the RMS amplitude of the baseline segment between the T‐ and P‐wave regions in the raw signal.

### Biocompatibility Tests

The cytotoxicity of the S‐LMC patch was evaluated using the Cell Counting Kit‐8 (CCK‐8) (Dojindo Laboratories, Kumamoto, Japan) assay with L‐929 mouse fibroblast cells (KCLB 10001, Korea). To specifically assess the biocompatibility of the liquid metal (LM) component, patch samples (square shape 1 cm^2^) were prepared by isolating the microchannel regions containing Galinstan. L‐929 cells were cultured in Dulbecco's modified Eagle medium (DMEM, Sigma‐Aldrich, USA) supplemented with 10% fetal bovine serum (FBS) and 1% penicillin‐streptomycin (PS) and maintained at 37 °C in a humidified atmosphere of 95% air and 5% CO_2_. The cell suspension, adjusted to 1 × 10^5^ cells/mL, was seeded into 96‐well plates and incubated for 24 h. After incubation, the medium was replaced with extract solution obtained from the S‐LMC patch after 24 h exposure to the culture medium. To validate the test, positive (0.1% ZDEC polyurethane film) and negative (planar PDMS) controls were included and treated under the same conditions using their respective extract solutions. After an additional 48 h incubation, CCK‐8 solution was added to each well at 10% of the total medium volume, followed by a 2 h incubation. Absorbance was measured at 450 nm using a microplate reader (SpectraMax Plus 384, Molecular Devices, USA).

### Institutional Review Board (IRB) Approval for Human Subject Research

All experiments involving human participants were conducted in accordance with the ethical guidelines and protocol approved by the Institutional Review Board of Ulsan National Institute of Science and Technology (UNISTIRB‐23‐061‐A). Written informed consent was obtained from all participants prior to their involvement in the study.

### Statistical Analysis

Statistical analyses were conducted on independently repeated experiments. The number of samples (n) for each dataset was specified in the corresponding figure panels. Datasets were analyzed using unpaired Student's t‐test for comparisons between two conditions, and one‐way ANOVA followed by Tukey's multiple comparison test was performed to compare three or more conditions. In all cases, P values less than 0.05 were considered statistically significant.

## Conflict of Interest

The authors declare no conflict of interest.

## Supporting information



Supporting Information

## Data Availability

The data that support the findings of this study are available from the corresponding author upon reasonable request.

## References

[1] a) J. Choi , A. J. Bandodkar , J. T. Reeder , T. R. Ray , A. Turnquist , S. B. Kim , N. Nyberg , A. Hourlier‐Fargette , J. B. Model , A. J. Aranyosi , S. Xu , R. Ghaffari , J. A. Rogers , ACS Sens. 2019, 4, 379; 30707572 10.1021/acssensors.8b01218

[2] a) G. Choi , J. Kim , H. Kim , H. Bae , B.‐J. Kim , H. J. Lee , H. Jang , M. Seong , S. M. Tawfik , J. J. Kim , H. E. Jeong , Adv. Mater. 2025, 37, 2412271; 39428834 10.1002/adma.202412271 PMC11775872

[3] a) Y. Cheng , Y. Zhou , R. R. Wang , K. H. Chan , Y. Liu , T. P. Ding , X.‐Q. Wang , T. T. Li , G. W. Ho , ACS Nano 2022, 16, 18608; 36318185 10.1021/acsnano.2c07097

[4] X. X. Lin , Z. P. Ou , X. W. Wang , C. Wang , Y. F. Ouyang , I. M. Mwakitawa , F. Li , R. Chen , Y. R. Yue , J. H. Tang , W. Fang , S. S. Chen , B. Guo , J. Y. Ouyang , T. Shumilova , Y. L. Zhou , L. Wang , C. W. Zhang , K. Sun , Interdiscip. Mater. 2024, 3, 775.

[5] Y. Gwon , S. Park , W. Kim , S. Park , H. Sharma , H. E. Jeong , H. Kong , J. Kim , Nano Lett. 2024, 24, 2188. 38324001 10.1021/acs.nanolett.3c04188

[6] a) Q. Q. Han , C. Zhang , T. M. Guo , Y. J. Tian , W. Song , J. X. Lei , Q. Li , A. H. Wang , M. L. Zhang , S. Bai , X. H. Yan , Adv. Mater. 2023, 35, 2209606; 10.1002/adma.202209606 36620938

[7] a) M. R. Carneiro , C. Majidi , M. Tavakoli , Adv. Funct. Mater. 2022, 32, 2205956;

[8] a) H. Jang , K. Sel , E. Kim , S. Kim , X. X. Yang , S. Kang , K.‐H. Ha , R. Wang , Y. F. Rao , R. Jafari , N. S. Lu , Nat. Commun. 2022, 13, 6604; 36329038 10.1038/s41467-022-34406-2 PMC9633646

[9] a) M. Abu Zahed , M. Sharifuzzaman , H. Yoon , M. Asaduzzaman , D. K. Kim , S. Jeong , G. B. Pradhan , Y. D. Shin , S. H. Yoon , S. Sharma , S. P. Zhang , J. Y. Park , Adv. Funct. Mater. 2022, 32, 2208344;

[10] a) R. Kusche , S. Kaufmann , M. Ryschka , Biomed. Phys. Eng. Express 2018, 5, 015001;

[11] a) M. A. Menke , B. M. Li , M. G. Arnold , L. E. Mueller , R. Dietrich , S. J. Zhou , N. Kelley‐Loughnane , P. Dennis , J. T. Boock , J. Estevez , C. E. Tabor , J. L. Sparks , Adv. Healthcare Mater. 2024, 13, 2301811; 10.1002/adhm.202301811 PMC11468510 37779336

[12] a) Y. Chen , Z. X. Liu , Z. G. Wang , Y. Yi , C. J. Yan , W. X. Xu , F. Zhou , Y. T. Gao , Q. T. Zhou , C. Zhang , H. Deng , Adv. Sci. 2024, 11, 2402759; 10.1002/advs.202402759 PMC11234450 38704681

[13] a) J. Jeon , J.‐W. Park , Nano Lett. 2024, 24, 9553; 39041723 10.1021/acs.nanolett.4c02107

[14] a) C. Qin , Q. Y. Sun , Y. Chen , S. Fahad , J. X. Wu , Y. X. Dong , H. Y. Yu , M. Wang , npj Flex. Electron. 2024, 8, 26;

[15] a) B. Peng , F. N. Zhao , J. F. Ping , Y. B. Ying , Small 2020, 16, 2002681; 10.1002/smll.202002681 32893485

[16] a) T. Li , H. B. Qi , C. C. Zhao , Z. M. Li , W. Zhou , G. J. Li , H. Zhuo , W. Zhai , Nat. Commun. 2025, 16, 88; 39747025 10.1038/s41467-024-55417-1 PMC11695986

[17] S. J. Yang , J. H. Cheng , J. Shang , C. Hang , J. Qi , L. N. Zhong , Q. Y. Rao , L. He , C. Q. Liu , L. Ding , M. M. Zhang , S. Chakrabarty , X. Y. Jiang , Nat. Commun. 2023, 14, 6494. 37838683 10.1038/s41467-023-42149-x PMC10576757

[18] a) C. Kim , J. Kim , J. Fan , M. Wang , F. Cicoira , Small Sci. 2025, 5, 2400553. 40395349 10.1002/smsc.202400553 PMC12087783

[19] S. Jamalzadegan , S. Kim , N. Mohammad , H. Koduri , Z. Hetzler , G. Lee , M. D. Dickey , Q. S. Wei , Adv. Funct. Mater. 2024, 34, 2308173.

[20] D. H. Ho , C. H. Hu , L. Li , M. D. Bartlett , Nat. Electron. 2024, 7, 1015.

[21] a) M. H. Duan , X. Y. Zhu , L. L. Fan , Y. Y. He , C. Yang , R. Guo , S. Chen , X. Y. Sun , J. Liu , Adv. Mater. 2022, 34, 2205002; 10.1002/adma.202205002 36018724

[22] a) H. Kim , G. Zan , Y. Seo , S. Lee , C. Park , Adv. Funct. Mater. 2024, 34, 2308703;

[23] T. Fang , Y. P. Sun , D. S. Kong , ACS Appl. Bio Mater. 2024, 7, 7791. 10.1021/acsabm.3c01298 38535705

[24] a) S. Park , D. K. Kang , D. Lee , G. Choi , J. Kim , C. Lee , M. Seong , M. D. Bartlett , H. E. Jeong , Sci. Adv. 2024, 10, adq3438; 10.1126/sciadv.adq3438 PMC11389778 39259793

[25] M. Seong , C. Park , J. Kim , M. Kim , J. Song , H. N. Kim , J. G. Ok , H. E. Jeong , Mater. Today Nano 2024, 27, 100488.

[26] a) J. Kim , G. Choi , S. Park , M. Kim , K. Kim , H. S. Jung , M. K. Kwak , J. G. Ok , H. E. Jeong , Int. J. Precis. Eng. Manuf. 2025, 26, 757;

[27] a) E. Arzt , S. Gorb , R. Spolenak , Proc. Natl. Acad. Sci. USA 2003, 100, 10603; 12960386 10.1073/pnas.1534701100 PMC196850

[28] a) S. Baik , H. J. Lee , D. W. Kim , J. W. Kim , Y. Lee , C. Pang , Adv. Mater. 2019, 31, 1803309; 10.1002/adma.201803309 30773697

[29] A. Tuteja , W. Choi , J. M. Mabry , G. H. McKinley , R. E. Cohen , Proc. Natl. Acad. Sci. USA 2008, 105, 18200. 19001270 10.1073/pnas.0804872105 PMC2587612

[30] X. L. Tian , V. Jokinen , J. Li , J. Sainio , R. H. A. Ras , Adv. Mater. 2016, 28, 10652. 27731514 10.1002/adma.201602714

[31] K. L. Johnson , Contact Mechanics, Cambridge University Press, Cambridge 1985.

[32] Z. L. Huang , Y. F. Hao , Y. Li , H. J. Hu , C. H. Wang , A. Nomoto , T. S. Pan , Y. Gu , Y. M. Chen , T. J. Zhang , W. X. Li , Y. S. Lei , N. Kim , C. F. Wang , L. Zhang , J. W. Ward , A. Maralani , X. S. Li , M. F. Durstock , A. Pisano , Y. Lin , S. Xu , Nat. Electron. 2018, 1, 473.

[33] Q. N. Zhuang , K. M. Yao , C. Zhang , X. Song , J. K. Zhou , Y. F. Zhang , Q. Y. Huang , Y. Z. Zhou , X. G. Yu , Z. J. Zheng , Nat. Electron 2024, 7, 598.

[34] K. Yamagishi , T. Ching , N. Chian , M. Tan , W. S. Zhou , S. Y. Huang , M. Hashimoto , Adv. Funct. Mater. 2024, 34, 1702589.

