# Supplementary material for: Self‐Adhesive Liquid Metal Channel Patch with Tip‐Guided Conformal Coupling and Leakage Suppression for Skin Bioelectronics
Source: Adv Sci (Weinh). 2025 Sep 12;13(1):e13259. doi: 10.1002/advs.202513259 (PMC12767012; doi:10.1002/advs.202513259)
Supplement: Supplementary file 1 — Supporting Information [file ADVS-13-e13259-s001.docx]

Supporting Information

Self-Adhesive Liquid Metal Channel Patch with Tip-Guided Conformal Coupling and Leakage Suppression for Skin Bioelectronics

Sang-Woo Lee, Hyeonseok Song, Jinseo Kim, Geonjun Choi, Jaeil Kim, Seongjin Park, Hyejin Jang, Jangho Kim^*^, and Hoon Eui Jeong^*^

S. -W. Lee, H. Song, J. Kim, G. Choi, J. Kim, S. Park, Prof. J. Kim, Prof. H. E. Jeong

Department of Mechanical Engineering

Ulsan National Institute of Science and Technology (UNIST)

Ulsan 44919, Republic of Korea

E-mail: hoonejeong@unist.ac.kr

Prof. J. Kim

Department of Convergence Biosystems Engineering

Chonnam National University

Gwangju 61186, Republic of Korea

E-mail: rain2000@jnu.ac.kr

**Note S1: Theoretical analysis of leakage suppression in re-entrant structures**

The confinement of liquid metal within re-entrant microchannels, such as the CPT structure of the S-LMC patch, is governed by the balance between internal pressure and surface tension, modulated by the local channel geometry. In particular, re-entrant geometries introduce strong pinning effects that resist interface deformation and delay the onset of leakage, in contrast to simple vertical wall designs like CP.

Based on the generalized force balance between the pressure inside the liquid (*P*) and the surface tension (*γ_lv_*), the condition for interface stability can be written as:^[S1]^

$$\begin{aligned} P\cdot A= \gamma_{lv}L\sin\delta\theta\end{aligned}$$

where *A* is the interfacial area and *L* is the contact line length.

On a textured surface, the local interface between liquid and vapor forms an intrinsic contact angle *θ* with the solid. As the applied pressure increases, the liquid-vapor interface becomes more severely distorted, and the degree of curvature can be described by the bulging angle *δθ*. Eventually, the local liquid-vapor interface reaches the tip boundary at the channel opening, where the local wall angle (*ψ*) reaches its minimum, as illustrated in Figure 4a. Any additional increase in pressure cannot be supported by changes in the local wall angle, resulting in the extrusion of the liquid metal through the microchannel opening and subsequent leakage. By re-writing the overall force balance, we can evaluate the theoretical interfacial pressure (*P_th_*) which is the pressure required to force a bulging angle of *δθ = θ – ψ_min_*.

$$\begin{aligned} 2\gamma_{lv}\sin\delta\theta=2\gamma_{lv}\sin\left( \theta-\psi_{min} \right)=D\cdot P_{th} \end{aligned}$$

$$\begin{aligned} P_{th}=\frac{{2\gamma}_{lv}\sin(\theta-\psi_{min})}{D} \end{aligned}$$

where *D* is the effective channel width and *ψ_min_* is the minimum local wall angle.

**Note S2: Theoretical description of reaction pressure using Hertzian contact theory**

In this study, an analytical estimation was employed to support the interpretation of the reaction pressure when a rigid circular cross-section is gradually embedded into a soft elastic substrate. Representative material properties were used for this estimation, with an elastic modulus representative of hydrated skin (*E* = 80 kPa) and a Poisson’s ratio (*ν* = 0.5), yielding a plane strain modulus (*E^*^ = E / (1 − ν²)* = 106,667 Pa).^[S2]^ According to the classic two-dimensional Hertzian contact theory for a rigid cylinder under plane strain conditions, the mean line pressure *P(δ)*, at an embedding depth *δ*, can be approximated as:^[S3]^

$$P(\delta)=\frac{\pi E^{*}}{4\sqrt{2}}\sqrt{\frac{\delta}{R}}$$

where *R* is the cylinder radius, defined as half of the effective channel width (*R = D/2*). As the embedding depth progressively increases from zero to *R* during loading, the average line pressure over the entire range, which represents the skin’s reaction pressure (*P_r_*), can be expressed as:

$$P_{r}=\frac{1}{R}\int_{0}^{R} P(\delta) d\delta=\frac{\pi E^{*}}{6\sqrt{2}}$$

For the given skin-like parameters, this yields a mean value of approximately 39.5 kPa. Although this approximation does not provide an exact solution for the specific experimental configuration, it serves as a practical reference for interpreting the local contact pressure behavior.

**Table S1.** Comparison of S-LMC patch and existing skin adhesives across adhesive, electrical performance and long-term stability.

| **Category** | **Skin adhesion strength (kPa)** | **Material** | **Contact impedance [kΩ cm^2^]** | **Long-term stability [day]** | **Refs** |
| --- | --- | --- | --- | --- | --- |
| S-LMC patch | 60.3 | Galinstan | 7.35 | 7 | This work |
| Metal | 18 * | Au | 150 * | 30 | [S4] |
| Hydrogel | 20 | PEDOT:PSS | 478.66 * | - | [S5] |
| Nanomaterial & Conductive polymers | 2.8 * | AgNW | 1986.1 * | - | [S6] |
| Nanomaterial & Conductive polymers | 70 | AgNW | 105.78 * | 7 | [S7] |
| Liquid metal | 9.6 * | Galinstan | 50 * | 7 | [S8] |
| Hydrogel | 6.5 * | rGO | 565.56 * | - | [S9] |
| Nanomaterial & Conductive polymers | 18.7 | MXene | 794 * | - | [S10] |
| Nanomaterial & Conductive polymers | 23 * | Au | 36 | - | [S11] |
| Hydrogel | 3 | Carbon Nanocomposite  (Graphite + Carbon black) | 8 * | - | [S12] |
| Nanomaterial & Conductive polymers | 90 * | Ag flake | 1550.97 * | 7 | [S13] |
| Hydrogel | 1.3 | CNT | 432 * | - | [S14] |
| Metal | 1.6 * | Au | 536.82 * | 1 | [S15] |
| Liquid metal | 15.2 | EGaIn | 6.375 * | - | [S16] |
| Nanomaterial & Conductive polymers | 3.4* | Au-NW | 61* | - | [S17] |
| Hydrogel | 9.5* | oligosulfobetaine methacrylate (OSBMA) | 17.17* | 10 | [S18] |
| Nanomaterial & Conductive polymers | 123* | PEDOT:PSS | 10.06* | - | [S19] |
| Metal | 20 | Mo-Au | 16* | 3 | [S20] |
| Liquid metal | 0.48* | EGaIn | 144.11 | - | [S21] |
| Nanomaterial & Conductive polymers | 16* | MXene/CNT | 455.93 | - | [S22] |
| Nanomaterial & Conductive polymers | 12.8* | PEDOT:PSS | 3449 | - | [S23] |
| Nanomaterial & Conductive polymers | 4.6* | PEDOT:PSS | 59 | - | [S24] |
| Nanomaterial & Conductive polymers | 2.9* | PEDOT:PSS | 7.15 | 11 | [S25] |

* This data is not directly provided by the corresponding references. Instead, we used the original data or extracted it from graphs to calculate the properties.
- This data is not provided by the corresponding references.


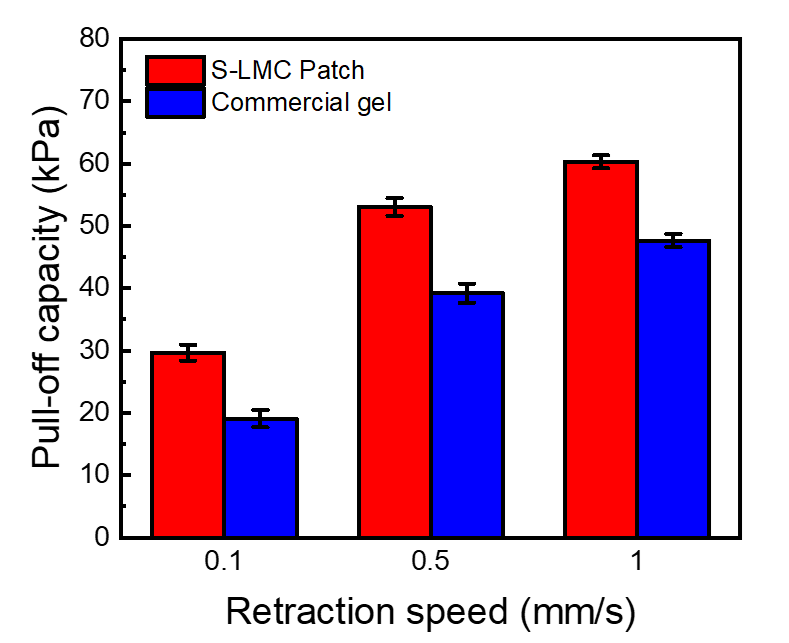


**Figure S1.** **Adhesion capacity of the S-LMC patch and commercial electrode under various retraction speeds, n = 7 independent samples.**


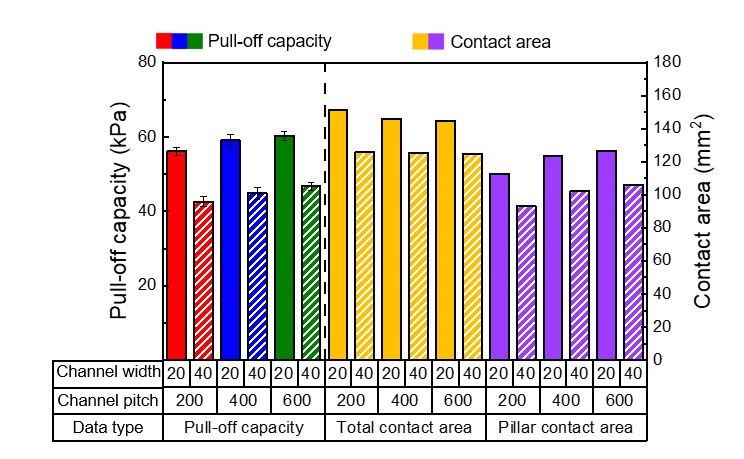


**Figure S2****. Pull-off capacity and contact area analysis of S-LMC patches with various microchannel geometries, n = 7 independent samples.**


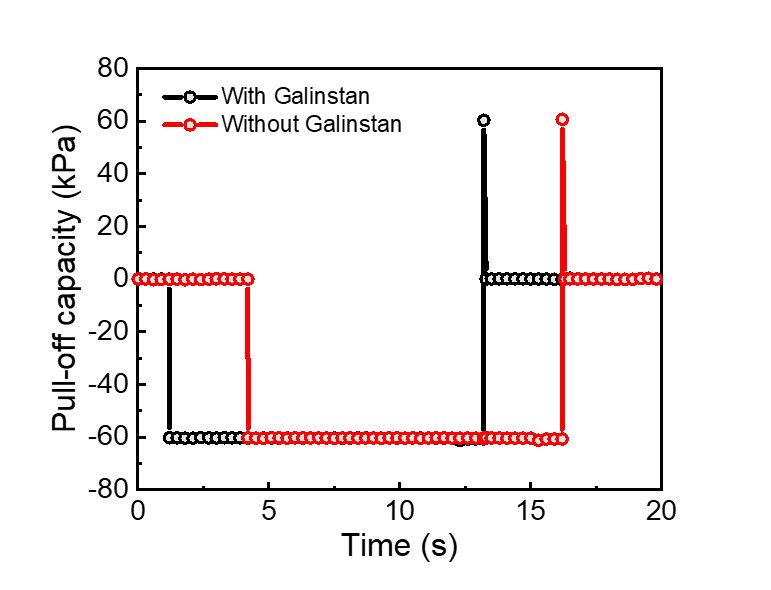


**Figure S3. Pull-off capacity of S-LMC patches with and without Galinstan.**


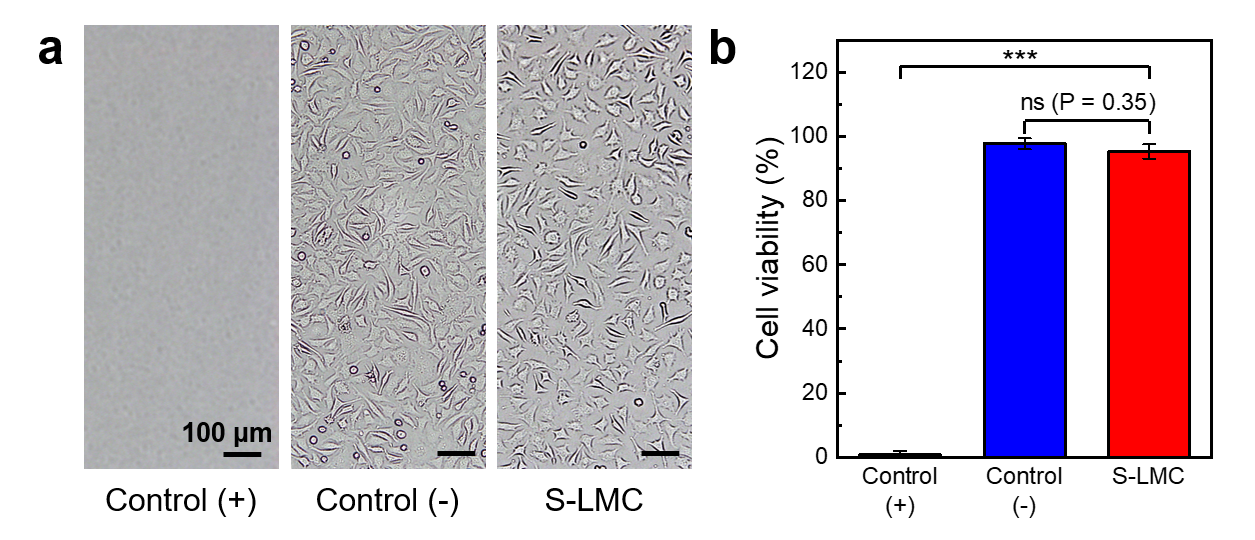


**Figure S4. In vitro cytotoxicity test of the S-LMC patch using murine fibroblasts after 48 h of culture. (a)** Microscopy images. **(b)** Quantitative analysis of cell viability, n = 5 independent samples.

**
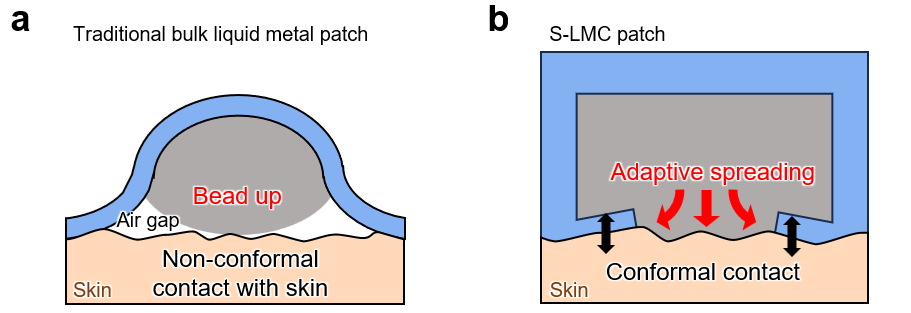
**

**Figure S5.** **Schematic comparison of the liquid metal wetting behavior on the skin. (a)** Bulk liquid metal. **(b)** S-LMC patch.

**
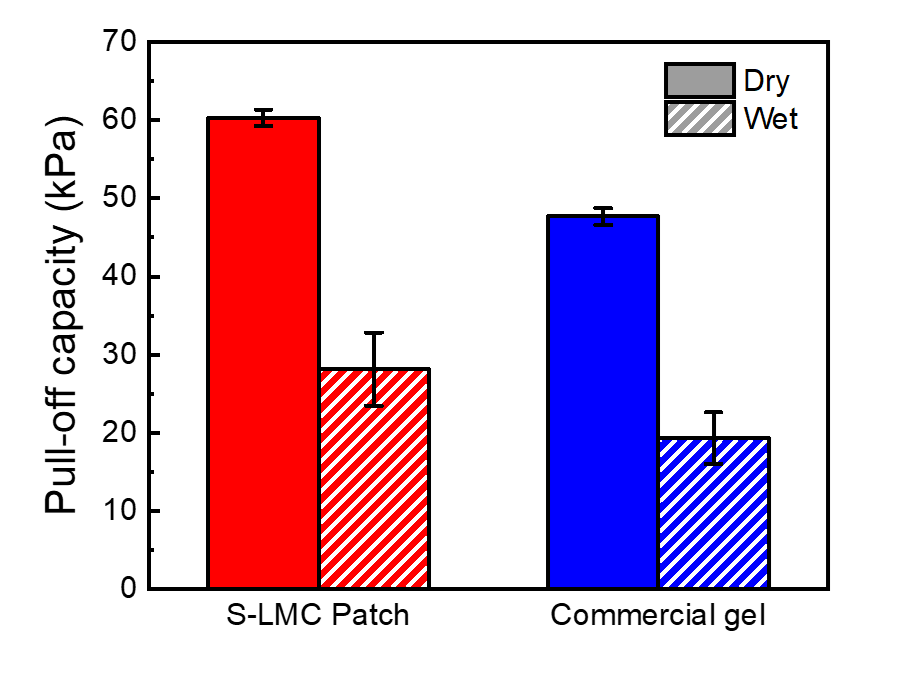
**

**Figure S6.** **Adhesion performance of the S-LMC patch and a commercial gel electrode under wet and dry conditions, n = 7 independent samples.**


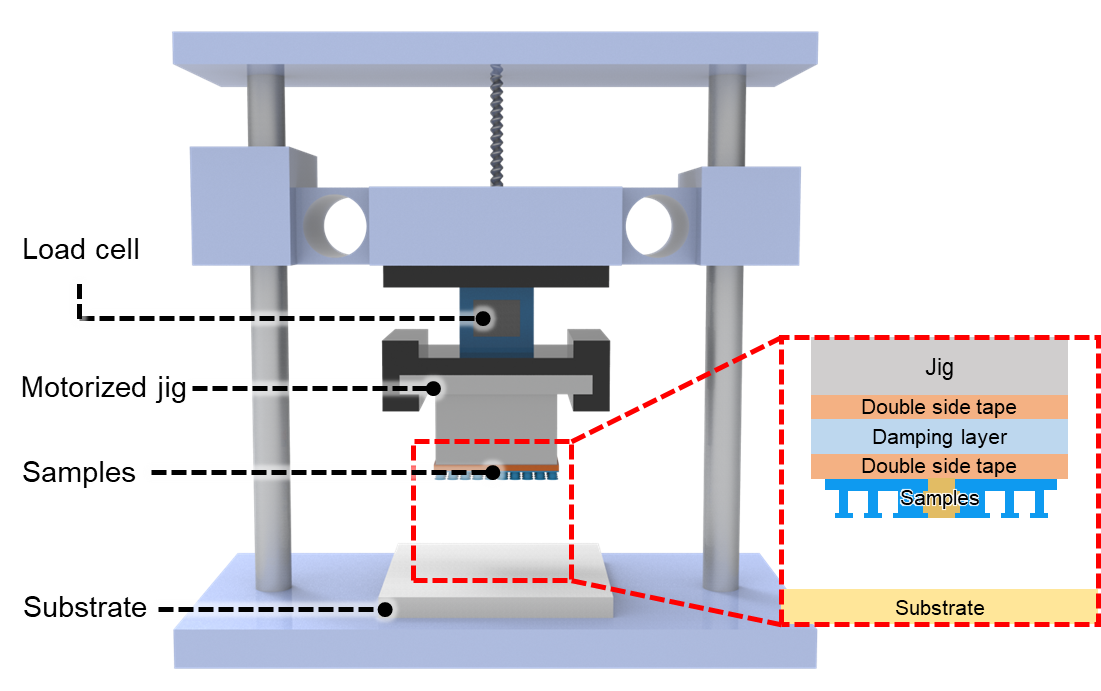


**Figure S7. Experimental setup employed for assessing the pull-off capacities of the adhesive samples.**

**References**

[S1] A. Tuteja, W. Choi, J. M. Mabry, G. H. McKinley, R. E. Cohen, *Proc. Natl. Acad. Sci. U.S.A.* **2008**, 105, 18200.

[S2] a) C. Pailler-Mattei, S. Bec, H. Zahouani, *Med. Eng. Phys.* **2008**, 30, 599; b) R. Sanders, *Pflugers Arch.* **1973**, 342, 255.

[S3] a) K. L. Johnson, *Contact Mechanics*, Cambridge University Press, Cambridge **1985**; b) J. M. Long, G. F. Wang, X. Q. Feng, S. W. Yu, *Int. J. Solids Struct.* **2012**, 49, 1588.

[S4] B. B. Zhang, J. Y. Li, J. K. Zhou, L. Chow, G. Y. Zhao, Y. Huang, Z. Q. Ma, Q. Zhang, Y. W. Yang, C. K. Yiu, J. Li, F. Chun, X. C. Huang, Y. Y. Gao, P. C. Wu, S. X. Jia, H. Li, D. F. Li, Y. M. Liu, K. M. Yao, R. Shi, Z. L. Chen, B. L. Khoo, W. Q. Yang, F. Wang, Z. J. Zheng, Z. K. Wang, X. E. Yu, *Nature* **2024**, 628, 84.

[S5] X. Zhou, A. Rajeev, A. Subramanian, Y. Li, N. Rossetti, G. Natale, G. A. A. Lodygensky, F. Cicoira, *Acta Biomater.* **2022**, 139, 296.

[S6] S.-R. Kim, J. Jeon, Y.-C. Kim, J.-W. Park, *Adv. Mater. Technol.* **2023**, 8, 2200968.

[S7] H. T. Lai, Y. Liu, Y. Cheng, L. J. Shi, R. R. Wang, J. Sun, *Adv. Sci.* **2023**, 10, 2300793.

[S8] T. X. Lan, H. M. Tian, X. L. Chen, X. M. Li, C. H. Wang, D. R. Wang, S. Li, G. Q. Liu, X. K. Zhu, J. Y. Shao, *Adv. Mater.* **2024**, 36, 2404761.

[S9] D. Kim, H. J. Lee, J. Oh, H. Y. Yang, H. J. Park, C. Huh, D. H. Ha, Y. Jun, Y. J. Yun, *J. Mater. Chem. C* **2025**, 13, 5711.

[S10] Y. F. Zhang, Z. S. Xu, M. K. Li, Y. Yuan, W. Wang, L. Q. Zhang, P. B. Wan, *Device* **2024**, 2, 100253.

[S11] Y. T. Huang, T. Araki, N. Kurihira, T. Kasuga, T. Sekitani, M. Nogi, H. Koga, *Adv. Mater. Interfaces* **2023**, 10, 2202263.

[S12] Y. Feng, H. Sun, M. Chen, C. Wu, J. K. Li, Z. Li, K. Makasheva, N. Liu, G. L. Zhang, W. J. Li, *IEEE Open J. Nanotechnol.* **2024**, 5, 156.

[S13] R. P. Zhang, S. Wen, Y. H. Zhao, S. B. Ji, *ACS Appl. Electron. Mater.* **2025**, 7, 925.

[S14] Z. Y. Wang, X. R. Xu, Y. T. Xu, W. E. Lin, Z. C. Peng, *npj Flex. Electron.* **2022**, 6, 51.

[S15] S. B. Ji, C. J. Wan, T. Wang, Q. S. Li, G. Chen, J. W. Wang, Z. Y. Liu, H. Yang, X. J. Liu, X. D. Chen, *Adv. Mater.* **2020**, 32, 2001496.

[S16] J. Kim, Y. Kim, J. Lee, M. Shin, D. Son, *Polymers* **2023**, 15, 3692.

[S17] H. Y. Zhang, Y. Y. Shen, L. X. Xing, W. Z. Wang, L. H. Liu, R. Jin, Z. Y. He, Y. T. Wang, S. H. Yao, X. Cao, S. R. Dong, B. W. Zhu, *Chem. Eng. J.* **2025**, 508, 160973.

[S18] K. He, P. Q. Cai, S. B. Ji, Z. H. Tang, Z. Fang, W. L. Li, J. Yu, J. T. Su, Y. F. Luo, F. L. Zhang, T. Wang, M. Wang, C. J. Wan, L. Pan, B. H. Ji, D. C. Li, X. D. Chen, *Adv. Mater.* **2024**, 36, 2311255.

[S19] C. Q. Li, Z. Y. Tan, X. H. Shi, D. K. Song, Y. Zhao, Y. Zhang, Z. H. Zhao, W. F. Zhang, J. Y. Qi, Y. F. Wang, X. Wang, Z. Q. Tan, N. Liu, *Adv. Sci.* **2024**, 11, 2406706.

[S20] X. Wang, C. S. Lu, Z. R. Jiang, G. W. Shao, J. Z. Cao, X. Y. Liu, *Adv. Sci.* **2025**, 12, 2410702.

[S21] M. A. Menke, B. M. Li, M. G. Arnold, L. E. Mueller, R. Dietrich, S. J. Zhou, N. Kelley-Loughnane, P. Dennis, J. T. Boock, J. Estevez, C. E. Tabor, J. L. Sparks, *Adv. Healthc. Mater.* **2024**, 13, 2301811.

[S22] Y. N. Hao, Q. Y. Yan, H. J. Liu, X. Y. He, P. H. Zhang, X. H. Qin, R. R. Wang, J. Sun, L. M. Wang, Y. Cheng, *Adv. Funct. Mater.* **2023**, 33, 2303881.

[S23] J. Jeon, J. W. Park, *Nano Lett.* **2024**, 24, 9553.

[S24] X. Lin, Z. Ou, X. Wang, C. Wang, Y. Ouyang, I. M. Mwakitawa, F. Li, R. Chen, Y. Yue, J. Tang, W. Fang, S. Chen, B. Guo, J. Ouyang, T. Shumilova, Y. Zhou, L. Wang, C. Zhang, K. Sun, *Interdiscip. Mater.* 3, 775.

[S25] S. J. Yang, J. H. Cheng, J. Shang, C. Hang, J. Qi, L. N. Zhong, Q. Y. Rao, L. He, C. Q. Liu, L. Ding, M. M. Zhang, S. Chakrabarty, X. Y. Jiang, *Nat. Commun.* **2023**, 14, 6494.
